# *TANC1::HTRA1* fusion in schwannomas

**DOI:** 10.1111/bpa.70084

**Published:** 2026-02-13

**Authors:** Ilay Caliskan, Kanish Mirchia, Walter Patrick Devine, Melike Pekmezci

**Affiliations:** ^1^ Department of Pathology, Division of Neuropathology University of California San Francisco San Francisco California USA; ^2^ Clinical Cancer Genomics Laboratory University of California San Francisco San Francisco California USA

Schwannomas are benign peripheral nerve sheath tumors that can arise either sporadically, frequently involving somatic alterations in *NF2*, *ARID1A*, *ARID1B*, and *DDR1* genes [1], or in the context of schwannomatosis, which is associated with germline *SMARCB1* or *LZTR1* mutations [2]. A distinct subset of schwannomas has been shown to harbor an *SH3PXD2A::HTRA1* fusion, including both sporadic cases [3] and those associated with *LZTR1* germline mutations [2]. These fusion‐positive tumors have a younger age of onset, preference for somatic sites over intracranial/spinal locations, and display a serpentine morphology [3]. Functional studies have demonstrated increased phosphorylated ERK (pERK) levels in vivo and in vitro [1], providing support for Ras/MEK/ERK pathway activation in *SH3PXD2A::HTRA1* fusion schwannomas. We describe two schwannomas harboring a fusion involving HTRA1 and an alternative partner, TANC1, which further supports that the HTRA1 portion of the fusion protein drives its tumorigenic activity.

Patient #1 is a 45‐year‐old male who presented with a palpable mass of the right medial upper thigh, found to have a 3 cm T2‐hyperintense heterogeneously enhancing tumor originating from the right obturator nerve on MR imaging (Figure 1A). Patient #2 is a 34‐year‐old female who presented with bilateral upper extremity paresthesia. MR imaging revealed a 4.5 cm homogenously enhancing tumor in the left C2–C3 neural foramen (Figure 1E).

**FIGURE 1:**
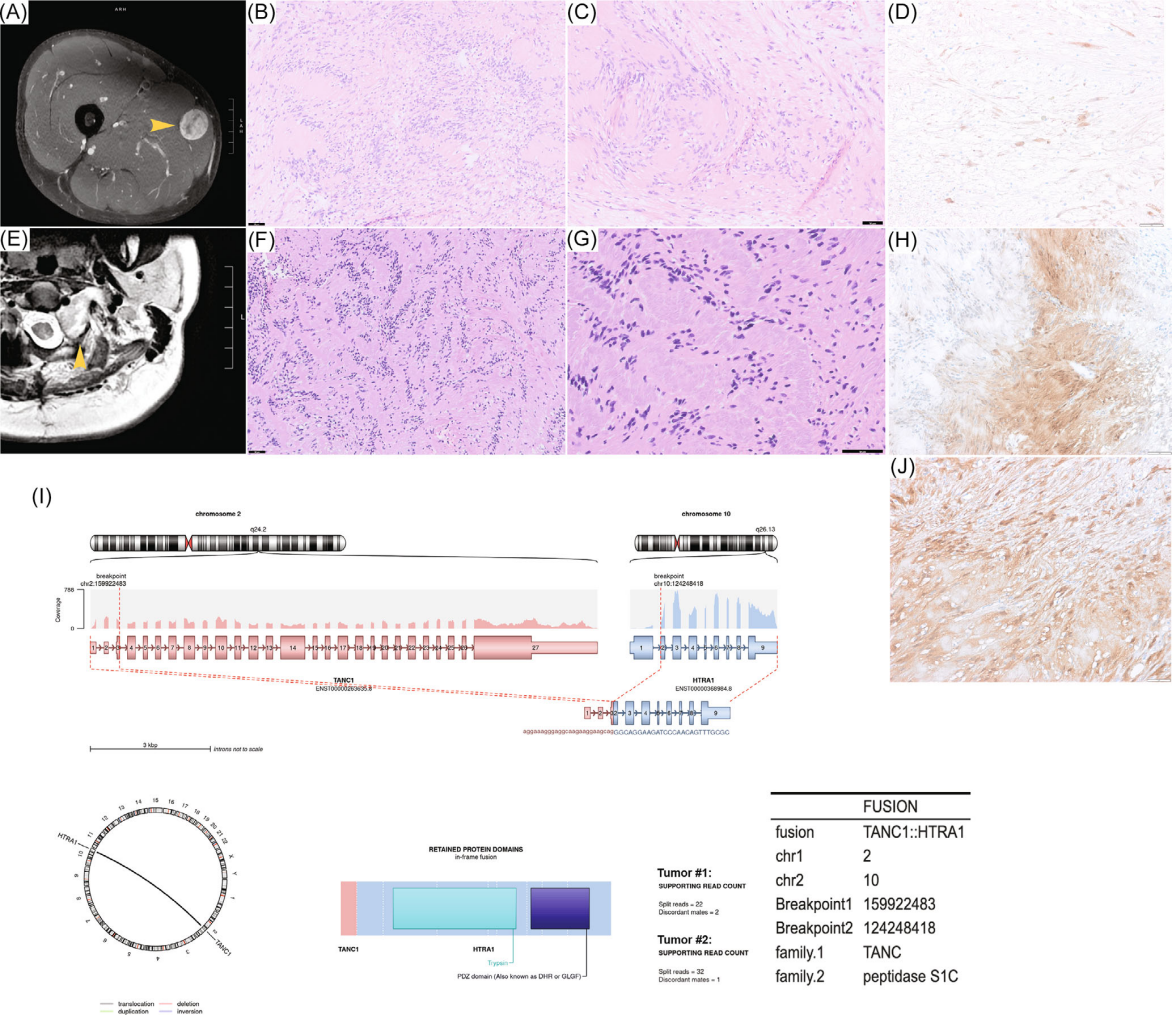
Radiologic, histologic, and genetic findings. (A) Axial T1‐weighted fat‐saturated post‐contrast image demonstrated tumor #1 (arrow), a well‐circumscribed 3 cm mass within the adductor longus and gracilis muscles, originating from the branches of right obturator nerve. (E) Axial T2‐weighted sequence showed tumor #2 (arrow), located in the left C2–C3 neural foramen extending into the spinal canal, resulting in cord compression. Histologic sections from tumor #1 (B, C), tumor #2 (F, G) show a variably cellular bland spindle cell neoplasm with fascicular arrangements focally forming twisting nuclear palisades and intervening areas of compact eosinophilic nuclear‐free zones, best illustrated in F (tumor #2). pERK immunohistochemistry showed patchy, predominantly cytoplasmic staining in both tumor #1 (D) and #2 (H), with comparable intensity to that of a schwannoma with *SH3PXD2A::HTRA1* fusion (J). Whole transcriptome RNA sequencing revealed an in‐frame *TANC1::HTRA1* fusion in both tumors (I).

The histologic sections from both tumors showed a variably cellular proliferation of bland spindle cells, arranged in fascicles and focally forming twisting nuclear palisades with intervening areas of compact eosinophilic nuclear‐free zones (Figure 1B,C,F,G), a pattern reminiscent but not classical for Verocay bodies. Mitotic activity was inconspicuous, with no cytologic atypia or necrosis.

Targeted DNA‐based next generation sequencing (NGS) was performed as previously described [4], which showed a solitary in‐frame gene fusion involving the *TANC1* and *HTRA1* genes as a result of a balanced translocation between chromosome 2q24 and chromosome 10q26 with breakpoints involving intron 3 of *TANC1* and intron 1 of *HTRA1* in tumor #1. Tumor #2 underwent a tumor‐normal paired targeted next‐generation sequencing which revealed no pathogenic or likely pathogenic somatic or germline alterations or copy number changes.

Whole transcriptome RNA sequencing demonstrated an in‐frame fusion (Figure 1I), where the N‐terminal portion is composed of exons 1–3 of *TANC1* and the C‐terminal is composed of exons 2–9 of *HTRA1*. The fusion proteins retain the functional trypsin and PDZ domains of the HTRA1, similar to what was described in the pathogenic *SH3PXD2A::HTRA1* fusion [3, 5].

*HTRA1* encodes a serine protease involved in the apoptosis, anoikis, and reorganization of extracellular matrix, and mechanistic studies showed its proteolytic activity depends on trimerization of activated HtrA1 monomers in a PDZ domain‐independent manner [6]. While *HTRA1* expression seems to be decreased in most cancers, the exact mechanism of *SH3PXD2A::HTRA1* fusion is unclear, beyond that the fusion protein promotes increased cell invasion and resistance to anoikis in a protease domain‐dependent manner [1].

TANC family proteins, including TANC1, are preferentially expressed in neural tissues and have been implicated in neuronal extension, differentiation, postsynaptic scaffolding [7], and myoblast fusion [8]. Rare structural variants involving *TANC1* have been previously described in salivary gland apocrine intraductal carcinomas with fusion transcripts including only the first four exons of *TANC1* [9]. Similarly, in our cases, both fusion transcripts included only the first 3 exons of this large gene and did not include any functional domains. Given the preferential expression of *TANC1* in neural tissues, and the fusion truncates the autoregulatory domains of Htra1, this rearrangement may also represent a promoter hijacking event.

To the best of our knowledge, there has been only one recent report of *TANC1::HTRA1* fusion in an intra‐abdominal schwannoma [10]. Although the initial cohort of *SH3PXD2A::HTRA1* fusion‐positive cases were predominantly intracranial, subsequent studies suggested preference for extracranial sites [3]. Similarly, our *TANC1::HTRA1* fusion‐positive cases were extracranial, and both tumors from this report demonstrate the typical twisting nuclear palisades as demonstrated by Lee et al. [3] Although the biological significance of this fusion remains uncertain without functional studies, we performed pERK immunohistochemistry (Cell Signaling; 4370S; dilution: 1:15,000) to assess for evidence of MEK/ERK pathway activation. Both tumors showed patchy, predominantly cytoplasmic pERK immunostaining (Figure 1D,H), with comparable intensity to that of an extracranial schwannoma with *SH3PXD2A::HTRA1* fusion (Figure 1J). This pattern suggests that the *TANC1::HTRA1* fusion may exert a tumorigenic effect similar to that of *SH3PXD2A::HTRA1*, thereby further supporting *HTRA1* as the driver gene and implicating MEK/ERK pathway inhibition as a potential therapeutic strategy [1].

## AUTHOR CONTRIBUTIONS

**MP** and **WPD** conceptualized the study, all authors contributed to data collection, analysis and interpretation, and writing and critical review of the manuscript.

## CONFLICT OF INTEREST STATEMENT

The corresponding author serves on the editorial board of this journal.

## Data Availability

Additional radiologic/histologic images and genomic data are available from the corresponding author upon request.
